# Iron Acquisition in Mycobacterium avium subsp. paratuberculosis

**DOI:** 10.1128/JB.00922-15

**Published:** 2016-02-12

**Authors:** Joyce Wang, Jalal Moolji, Alex Dufort, Alfredo Staffa, Pilar Domenech, Michael B. Reed, Marcel A. Behr

**Affiliations:** a Department of Microbiology and Immunology, McGill University, Montreal, Quebec, Canada; b McGill University and Genome Quebec Innovation Centre, Montreal, Quebec, Canada; c Research Institute of the McGill University Health Centre, Montreal, Quebec, Canada; d McGill International TB Centre, Montreal, Quebec, Canada; e Department of Medicine, McGill University, Montreal, Quebec, Canada

## Abstract

Mycobacterium avium subsp. paratuberculosis is a host-adapted pathogen that evolved from the environmental bacterium M. avium subsp. hominissuis through gene loss and gene acquisition. Growth of M. avium subsp. paratuberculosis in the laboratory is enhanced by supplementation of the media with the iron-binding siderophore mycobactin J. Here we examined the production of mycobactins by related organisms and searched for an alternative iron uptake system in M. avium subsp. paratuberculosis. Through thin-layer chromatography and radiolabeled iron-uptake studies, we showed that M. avium subsp. paratuberculosis is impaired for both mycobactin synthesis and iron acquisition. Consistent with these observations, we identified several mutations, including deletions, in M. avium subsp. paratuberculosis genes coding for mycobactin synthesis. Using a transposon-mediated mutagenesis screen conditional on growth without myobactin, we identified a potential mycobactin-independent iron uptake system on a M. avium subsp. paratuberculosis-specific genomic island, LSP^P^15. We obtained a transposon (Tn) mutant with a disruption in the LSP^P^15 gene *MAP3776c* for targeted study. The mutant manifests increased iron uptake as well as intracellular iron content, with genes downstream of the transposon insertion (*MAP3775c* to *MAP3772c* [*MAP3775-2c*]) upregulated as the result of a polar effect. As an independent confirmation, we observed the same iron uptake phenotypes by overexpressing *MAP3775-2c* in wild-type M. avium subsp. paratuberculosis. These data indicate that the horizontally acquired LSP^P^15 genes contribute to iron acquisition by M. avium subsp. paratuberculosis, potentially allowing the subsequent loss of siderophore production by this pathogen.

**IMPORTANCE** Many microbes are able to scavenge iron from their surroundings by producing iron-chelating siderophores. One exception is Mycobacterium avium subsp. paratuberculosis, a fastidious, slow-growing animal pathogen whose growth needs to be supported by exogenous mycobacterial siderophore (mycobactin) in the laboratory. Data presented here demonstrate that, compared to other closely related M. avium subspecies, mycobactin production and iron uptake are different in M. avium subsp. paratuberculosis, and these phenotypes may be caused by numerous deletions in its mycobactin biosynthesis pathway. Using a genomic approach, supplemented by targeted genetic and biochemical studies, we identified that LSP^P^15, a horizontally acquired genomic island, may encode an alternative iron uptake system. These findings shed light on the potential physiological consequence of horizontal gene transfer in M. avium subsp. paratuberculosis evolution.

## INTRODUCTION

Mycobacterium avium subsp. paratuberculosis is the causative agent of Johne's disease, also known as paratuberculosis, a chronic gastrointestinal inflammation in ruminants (1, 2). Since the early days of M. avium subsp. paratuberculosis science, researchers have noted its inability to grow in the laboratory in the absence of extracts from other mycobacteria such as M. tuberculosis and M. phlei (2). Not until 40 years later was the “growth factor” isolated from M. phlei and the term “mycobactin” (MBT) coined (3). Mycobactins have subsequently been elucidated to be iron (Fe)-binding siderophores, produced by various saprophytic and pathogenic mycobacteria (4–6).

The synthesis, structure, and utilization of mycobactins have been extensively studied in M. tuberculosis, the etiological agent of human tuberculosis (7–10). The synthesis of M. tuberculosis mycobactin (mycobactin T) requires two genetic clusters, *mbt-1* (*mbtA* to *mbtJ*) and *mbt-2* (*mbtK* to *mbtN*), encoding 14 nonribosomal peptide synthases (NRPSs) (11, 12). These enzymes catalyze the conversion of chorismate into salicylic acid, which ultimately gives rise to mycobactin, a structurally complex compound containing a high-affinity binding site for ferric iron (Fe^3+^) (7, 8). Notably, while mycobactin is lipophilic due to the presence of a long alkyl chain, its solubility in aqueous solutions can be increased by replacing the long alkyl chain with a shorter chain terminating in a carboxylic acid group, and the modified compound is called “carboxymycobactin” (cMBT). The hydrophobic form of mycobactin is cell associated, and the hydrophilic form is secreted (8). Because of their role in iron uptake, mycobactins are important for the survival and replication of M. tuberculosis inside the host (8, 13, 14).

Like M. tuberculosis, M. avium subsp. paratuberculosis is an intracellular pathogen that encounters a nutritionally stringent host environment, deprived of important nutrients, including iron (15–17). It is thus an intriguing phenomenon that M. avium subsp. paratuberculosis growth *in vitro* is mycobactin dependent and cannot be promoted by animal tissue or host iron-binding proteins. These findings can be interpreted to indicate that M. avium subsp. paratuberculosis acquires iron in a mycobactin- and host-independent fashion (2, 18). Whole-genome sequencing of a common clone (K10) revealed that the homolog of *mbtA*, encoding the first enzyme acting on salicylic acid in the mycobactin biosynthesis pathway, is truncated (19), potentially inactivating mycobactin production (7). These genetic and phenotypic features altogether argue that an alternative iron uptake system(s) may be encoded by the genome for M. avium subsp. paratuberculosis to survive *in vivo* (18, 20, 21).

Genomic comparisons indicate that M. avium subsp. paratuberculosis, a host-adapted pathogen, arose from an environmental organism, M. avium subsp. hominissuis (22). Compared to M. avium subsp. hominissuis, M. avium subsp. paratuberculosis strains have reduced genomes and decreased genetic variability (19, 22), with two lineages, “cattle” and “sheep” (referred to herein as MAP-cattle and MAP-sheep, respectively), defined by lineage-specific genomic features (23). In spite of having a smaller genome, M. avium subsp. paratuberculosis has 96 additional genes distributed across 6 genomic islands (also known as large sequence polymorphisms [LSP^P^s]): LSP^P^4, LSP^P^11, LSP^P^12, LSP^P^14, LSP^P^15, and LSP^P^16. As these islands are absent in other mycobacteria, including M. avium subsp. hominissuis, it has been proposed that they were acquired via horizontal gene transfer prior to the emergence of these two sublineages of M. avium subsp. paratuberculosis (24). Two of these M. avium subsp. paratuberculosis-specific islands, LSP^P^14 and LSP^P^15, are predicted to encode several metal uptake systems. For example, LSP^P^15 (5.4 kb) is predicted to encode an ATP-binding cassette (ABC) transporter (MAP3776-4c), a metal uptake regulator (regulating ferric iron or zinc; abbreviated FurB or Zur, respectively)-like transcription factor (MAP3773c), and a product that may be involved in cobalamin (vitamin B_12_) synthesis (MAP3772c) (24). In this report, we revisit mycobactin dependence in M. avium subsp. paratuberculosis and investigate whether a potential alternative iron acquisition mechanism(s) is encoded in the M. avium subsp. paratuberculosis-specific regions of its genome.

## MATERIALS AND METHODS

### Culture conditions and chemicals.

All mycobacterial strains were grown in Middlebrook 7H9 broth (Difco Laboratories, Detroit, MI) containing 0.2% glycerol, 0.1% Tween 80, and 10% albumin-dextrose-catalase (Becton, Dickinson and Co., Sparks, MD) with rotation at 37°C. Middlebrook 7H10 solid medium supplemented with 10% oleic acid-albumin-dextrose-catalase (Becton, Dickinson and Co., Sparks, MD) was used to isolate single colonies. The 7H9 and 7H10 media were supplemented with mycobactin J (mJ) (Allied Monitor, IN) as indicated. Iron-depleted glycerol-alanine salts with Tween 80 (GAST) medium was prepared using Chelex 100 resin (Bio-Rad) (10 g/liter) for the mycobactin production and radioactive iron uptake assays (8). Chemically competent Escherichia coli (New England BioLabs) was grown in Luria-Bertani medium (Difco) at 37°C. Antibiotics were added when needed as follows: apramycin at 60 μg/ml for E. coli; hygromycin at 100 μg/ml for E. coli and 50 μg/ml for mycobacteria; and kanamycin at 50 μg/ml for both E. coli and mycobacteria. All chemicals used were purchased from Sigma-Aldrich (St. Louis, MO) unless stated otherwise.

### Mycobactin detection assay.

Low-iron GAST medium was prepared as described above. M. smegmatis, M. tuberculosis, M. avium subsp. avium, M. intracellulare, M. avium subsp. hominissuis, MAP-cattle (laboratory strain K10, field isolate Cow69, and attenuated vaccine strain 316F), and MAP-sheep field isolates (6756S and P465) were grown in low-iron GAST medium in the presence of 0.5 μCi/ml of 7-^14^C salicylic acid for 11 days (except M. smegmatis, which was grown overnight). Water-soluble and cell-associated mycobactins were extracted with chloroform, spotted onto silica gel 60 thin-layer chromatography (TLC) plates (EMD Millipore), and developed in hexane/butanol/ethyl acetate (2:3:3) as described previously (8). TLC plates were visualized using a Storm 840 PhosphorImager (GE Healthcare).

### Iron uptake assays.

Iron uptake assays were performed as previously described with minor modifications (25). All strains were grown in 7H9 broth without mycobactin J, transferred to GAST medium and grown for 4 days to reduce any residual iron/mycobactin, and then washed twice with GAST medium and grown in fresh GAST medium. After 3 days, all cultures were adjusted to an optical density at 600 nm (OD_600_) of 0.1 to 0.15, and 0.5 μCi/ml of ^55^FeCl_3_ (American Radiolabeled Chemicals, St. Louis, MO) was added. At the indicated times, the OD_600_ was measured and 2 ml of culture was collected for intracellular ^55^FeCl_3_ quantification in triplicate. Samples were centrifuged for 15 min at 13,500 rpm, and the pellets were washed twice with phosphate-buffered saline (PBS) with 0.05% Tween 80, resuspended in 100 μl of PBS-Tween, and transferred to 20 ml of Ultima Gold scintillation cocktail (Perkin-Elmer). Radioactivity was then measured for 5 min in the ^55^Fe channel of a Tri-Carb 3110TR scintillation counter (Perkin-Elmer). ^55^FeCl_3_ accumulation is presented as counts per minute/OD_600_.

### Intracellular metal quantification.

M. avium subsp. paratuberculosis strains were grown in triplicate, subjected to different (namely, 0%, 0.1%, and 1.0% [wt/vol]) ferric ammonium citrate (FAC) supplementations, and harvested 48 h after inoculation. Total iron (Fe) and magnesium (Mg) levels were determined by inductively coupled plasma mass spectrometry (ICP-MS; Concordia University, Montreal, Quebec, Canada) as described elsewhere (26). Briefly, bacterial pellets were washed twice in Optima water (Fisher Scientific), transferred to acid-washed Falcon tubes (Corning), digested with 65% nitric acid at 80°C for 1 h, and diluted with trace-analysis-grade water to a final concentration of 2% nitric acid. All plastic consumables that came in contact with nitric acid were acid washed prior to experiments (27). The abundance of each metal was quantified using an external standard, and the intracellular Fe:Mg ratio of each sample was calculated. Raw data are provided in Table S2 in the supplemental material.

### Transposon insertion mutant library screening.

M. avium subsp. paratuberculosis K10 (cattle lineage) was used as the parental strain in this study. Bacteria were grown and two independent transposon mutant libraries were generated as described previously (28). Glycerol stocks of two libraries were plated on agar plates containing kanamycin with or without mycobactin J supplementation. To minimize the effect of the presence of mycobactin carried over from previous culturing, 10^5^ CFU were collected under each set of conditions and were replated under the same conditions. Genomic DNA was extracted from these libraries at the second passage. The 4 libraries (library 1 and 2 and the same libraries with and without mycobactin J) were processed for transposon sequencing (29). The libraries were indexed and sequenced on the Illumina HiSeq 2000 platform at the McGill University and Génome Québec Innovation Centre, and 100-bp reads were generated. The transposon sequence (CGGGGACTTATCAGCCAACCTGT) was trimmed using cutadapt (30). The genetic requirement under each condition (with or without mycobactin J) was computationally analyzed as described previously (28).

In a separate transposon library, a collection of ∼5,000 transductants was screened for transposon insertions in LSP^P^15 by PCR using a universal forward primer that binds to the flanking inverted repeats within the transposon sequence and multiple reverse primers annealing to different genes near or within the LSP^P^15 locus. Genomic DNA was extracted from PCR-positive clones and digested with either BamHI or SacII (New England BioLabs), and the excised fragment was subsequently circularized and cloned into a *pir*-positive (*pir*^+^) E. coli strain for plasmid extraction and Sanger sequencing at the McGill University and Génome Québec Innovation Centre. All primers used in the study are listed in Table S1 in the supplemental material. All primers were designed using Primer3 (31) unless specified otherwise.

### RNA isolation and quantitative RT-PCR (qRT-PCR).

RNA was extracted from cultures at an OD_600_ of 0.3 to 0.5 and reverse transcribed (RT) into randomly primed first-strand cDNA as previously described (32). Briefly, cell pellets were resuspended in 1 ml of TRIzol (Invitrogen) and were mechanically disrupted with 0.1-ml-diameter glass beads using a homogenizer (MP Biomedicals) with 3 pulses at maximum speed (6.0) for 30 s with a 3-min incubation on ice between pulses. The TRIzol phase was then transferred and vigorously mixed with 200 μl of chloroform-isoamyl alcohol (24:1). Following centrifugation, the upper aqueous phase was added to 500 μl isopropanol to precipitate the RNA. After washing with 70% ethanol was performed, the RNA pellet was treated with Turbo DNase (Life Technologies) and cleaned up using an RNeasy kit (Qiagen), followed by two Turbo DNase treatments. First-strand cDNA synthesis was performed using a RevertAid first-strand cDNA synthesis kit following the manufacturer's recommendation (Life Technologies). Conventional PCR using *sigA* primers was performed for all RNA and cDNA samples to verify the absence of genomic DNA contamination and successful cDNA synthesis, respectively. Samples were measured in triplicate to assess the expression of each gene in the locus and normalized to *sigA* as an endogenous control. The reagents, instrumentation, and data analysis used are described in reference 33, and primers were designed using Primer Express 3.0 (Applied Biosystems). Gene expression of wild-type M. avium subsp. paratuberculosis and the transposon mutant was measured in at least three independent experiments.

### PCR-based transcription mapping and 5′ rapid amplification of cDNA ends (RACE).

To determine if LSP^P^15 genes are cotranscribed, we amplified sequences flanking the junction of adjacent genes using wild-type M. avium subsp. paratuberculosis cDNA as the template. To examine the transcription start site (TSS) of the first gene, *MAP3776c*, we identified multiple putative start codons (GTG or ATG) at the 5′ side of the gene and amplified each segment to determine where transcription starts.

As a complementary approach to determine the transcription start site, 5′ RACE was performed using wild-type M. avium subsp. paratuberculosis K10 total RNA, following the manufacturer's instructions (GeneRacer; Invitrogen). Due to the nature of prokaryotic mRNA, dephosphorylation by calf intestinal phosphatase was omitted in our procedure. RNA was decapped with tobacco acid pyrophosphatase and ligated to the RNA oligonucleotide provided. Reverse transcription was carried out using random hexamer primers and Superscript RT III enzyme. GeneRacer 5′ primer and *MAP3776c*-specific primers were used to amplify a region flanked by the RNA oligonucleotide and part of *MAP3776c* close to the 3′ end. A nested PCR was subsequently carried out. Both PCRs were performed using Phusion high-fidelity DNA polymerase (New England BioLabs) at an annealing temperature of 65°C. The amplified fragment was then cloned into a TOPO vector for blunt-end PCR products (Invitrogen) and sequenced with M13 primers.

### Overexpression of LSP^P^15 genes.

Wild-type M. avium subsp. paratuberculosis K10 was used as the parental strain for overexpressing *MAP3775-2c*, following the procedures described (34). Briefly, *MAP3775-2c* was PCR amplified using Phusion high-fidelity DNA polymerase and cloned into pMV261-apramycin, an episomal vector, with the gene positioned directly downstream of a constitutive *hsp60* promoter. The *hsp60-MAP3775-2c* fragment was subsequently excised with SpeI and HindIII and shuttled into pMV306-hygromycin, an integrative vector. After transformation into E. coli, plasmids were purified using a QIAprep Spin Miniprep kit (Qiagen), confirmed with restriction digestion and Sanger sequencing, and subsequently electroporated into wild-type M. avium subsp. paratuberculosis. M. avium subsp. paratuberculosis was rendered electrocompetent using a series of 10% glycerol washes at room temperature (35). Transformants were picked from 7H10 plates and grown in 7H9 liquid broth supplemented with mycobactin J and hygromycin. Successful integration was confirmed by PCR, and gene expression was validated using quantitative RT-PCR (qRT-PCR).

### Statistical analysis.

Data are presented as means ± standard deviations of the means (analyzed by the Student *t* test), and variances between samples were determined by the F-test.

## RESULTS

### Exogenous mycobactin promotes iron accumulation and growth in M. avium subsp. paratuberculosis.

**FIG 1:**
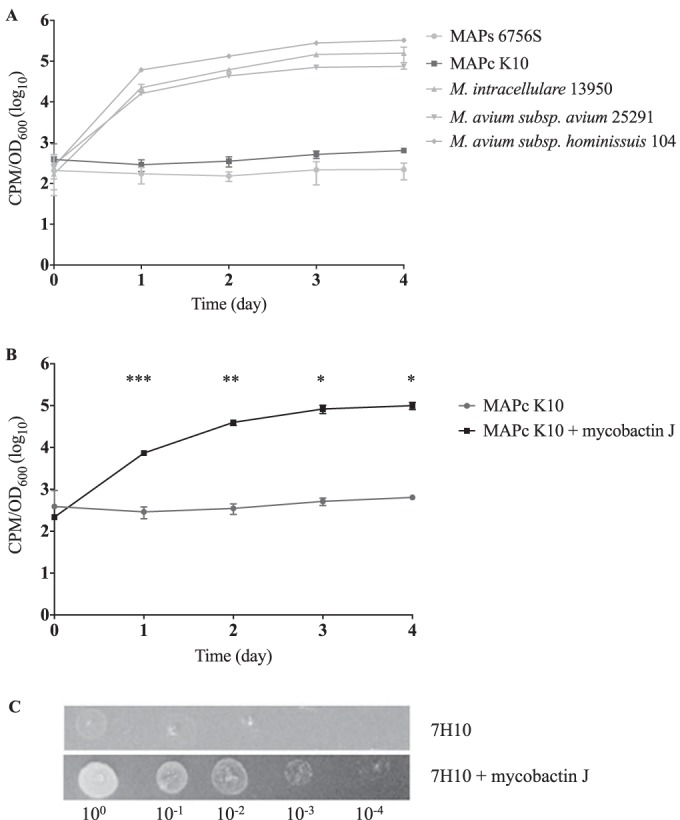
M. avium subsp. paratuberculosis *in vitro* “dependence” on mycobactin. (A) ^55^FeCl_3_ uptake assay in MAP-cattle (MAPc), MAP-sheep (MAPs), and several other M. avium subspecies. (B) ^55^FeCl_3_ uptake assay in MAP-cattle in the presence or absence of exogenous mycobactin J. *, *P* < 0.05; **, *P* < 0.01; ***, *P* < 0.001. (C) MAP-cattle growth assay in the presence or absence of exogenous mycobactin J on 7H10 agar. Tenfold serial dilutions of wild-type M. avium subsp. paratuberculosis K10 grown in 7H9 without mycobactin J supplementation were spotted onto 7H10 with or without mycobactin J. The plates were incubated at 37°C for 6 weeks and then photographed.

Iron uptake was assessed in M. avium subsp. paratuberculosis K10 (cattle lineage) and 6756S (sheep lineage), as well as in several M. avium subspecies, including M. avium subsp. hominissuis, M. avium subsp. *avium*, and M. intracellulare. These strains were pregrown in iron-depleted GAST medium treated with the iron-scavenging Chelex resin, and their ability to accumulate iron was subsequently quantified by monitoring uptake of ^55^FeCl_3_. Over 4 days, the iron content increased by 2 to 3 logs in M. avium subsp. hominissuis, M. avium subsp. *avium*, and M. intracellulare (Fig. 1A), in striking contrast to the M. avium subsp. paratuberculosis cultures. When M. avium subsp. paratuberculosis K10 culture was supplemented with 2 μg/ml of commercial mycobactin J (mJ), accumulation of radiolabeled iron from the culture media increased by 460-fold on day 4 compared to the mJ-free culture results (Fig. 1B). When M. avium subsp. paratuberculosis K10 was grown on 7H10 agar, the supplementation of mJ visibly supported growth to a much greater extent after 6 weeks of incubation (Fig. 1C). The results of these experiments clearly indicate that M. avium subsp. paratuberculosis strains are defective in iron uptake and that this defect can be reversed in the presence of exogenous mycobactin.

### Different mycobactin production patterns in M. avium subsp. paratuberculosis strains.

**FIG 2:**
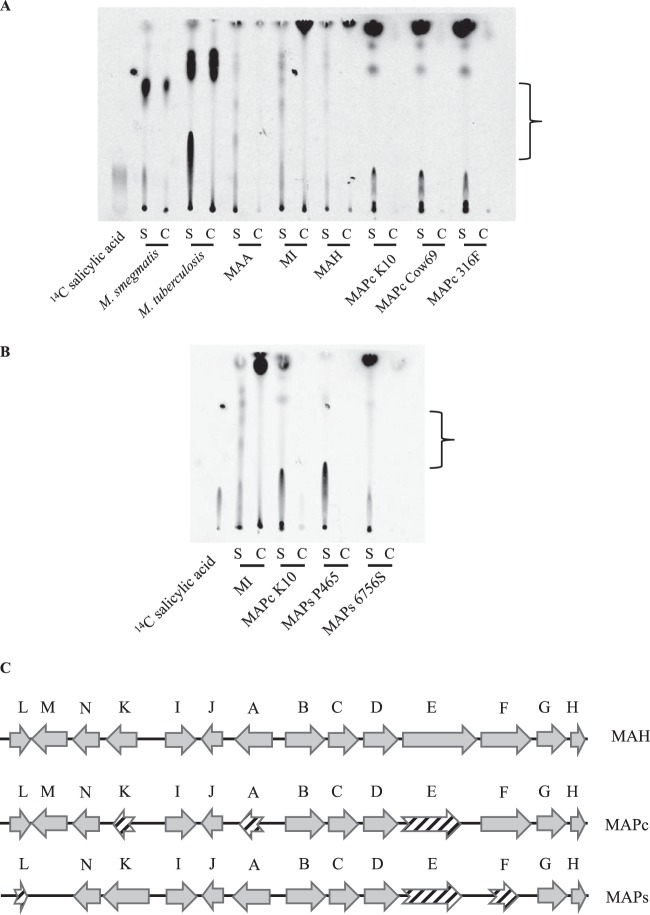
Investigation of mycobactin production and biosynthetic pathway. (A) TLC of mycobactins extracted from various mycobacterial cultures labeled with 7-^14^C salicylic acid. The bracketed region includes labeled species present in M. smegmatis, M. tuberculosis, and M. avium species but not M. avium subsp. paratuberculosis. S, secreted (medium); C, cell associated; MAA, M. avium subsp. *avium*; MI, M. intracellulare; MAH, M. avium subsp. hominissuis. (B) TLC of mycobactins extracted from M. intracellulare, MAP-cattle K10, and MAP-sheep S6756 and P465. The bracketed region includes labeled species present in M. intracellulare species but not M. avium subsp. paratuberculosis. S, secreted (medium); C, cell associated. (C) Organization of the *mbt* clusters in M. avium subsp. hominissuis, MAP-cattle (MAPc), and MAP-sheep (MAPs). Striped arrows represent protein products with less than 80% amino acid similarity.

To investigate whether the impairment in iron uptake is associated with the inability to produce mycobactin in M. avium subsp. paratuberculosis, we extracted both secreted carboxymycobactin (cMBT) and cell-associated mycobactin (MBT) from various mycobacterial strains grown in iron-depleted GAST medium. M. smegmatis, a saprophytic species known to produce cMBT and MBT (36, 37), and M. tuberculosis contained material strongly labeled with ^14^C salicylic acid in both the culture filtrate and cell pellet fractions. M. avium subsp. *avium*, M. intracellulare, and M. avium subsp. hominissuis also secreted several radiolabeled species in the culture filtrate that showed similar retention factor (Rf) values with respect to the major species identified in M. tuberculosis, albeit at a reduced relative abundance. In contrast, the majority of these species were completely absent in the 3 MAP-cattle strains examined (Fig. 2A); instead, these produced a TLC profile that was completely distinct from that observed for the other M. avium subspecies examined. We then explored whether the same pattern was observed in MAP-sheep strains, with the same deficiency in mycobactin production seen (Fig. 2B). Inspection of the sequences of all *mbt* genes (*mbtA* to *mbtK*) in M. avium subsp. hominissuis, MAP-cattle (K10 genome), and MAP-sheep (S397 genome [38]) revealed numerous mutations restricted to MAP-cattle or MAP-sheep, several leading to putative protein truncations (Fig. 2C). Notably, the reference strains of MAP-cattle and -sheep share a 31-bp deletion in the DNA sequence corresponding to M. avium subsp. hominissuis *mbtE* (position 5053 to position 5084), resulting in a premature stop codon, with MAP-sheep having incurred an additional 152-bp deletion in the *mbtE* gene (position 3778 to position 3930 in M. avium subsp. hominissuis *mbtE*) (Fig. 2C; see also Fig. S1 in the supplemental material). From these analyses, one possibility that emerged is that the common ancestor of these sequenced M. avium subsp. paratuberculosis genomes had the 31-bp *mbtE* deletion, following which other mutations in the mycobactin gene cluster accumulated in MAP-cattle and MAP-sheep.

### LSP^P^15 disruption adversely affects M. avium subsp. paratuberculosis fitness in the absence of mycobactin.

Based on phenotypic, biochemical, and *in silico* analyses, mycobactin biosynthesis appears to be defective in both MAP-cattle and -sheep. We subsequently explored whether the M. avium subsp. paratuberculosis genome encodes another, mycobactin-independent iron uptake system(s) that might obviate the need to make mycobactin. To investigate the genetic requirement for M. avium subsp. paratuberculosis growth in a mycobactin-free environment, we employed a transposon-mediated mutagenesis approach to compare mutant abundance in the presence and absence of mycobactin J. We sequenced two independently constructed M. avium subsp. paratuberculosis K10 transposon libraries to identify genes that, when disrupted, resulted in mutants that were depleted (5× less than the median) in the absence of mycobactin J (Table 1; see also Table S3 in the supplemental material). One of the genes so identified, in both libraries, was *MAP3775c*, situated in a M. avium subsp. paratuberculosis-specific genomic island, LSP^P^15. By bioinformatic analysis, LSP^P^15 (5.4 kb) is predicted to encode an ATP-binding cassette (ABC) transporter (MAP3776-4c), a metal uptake regulator (MAP3773c), a protein that may be involved in cobalamin (vitamin B_12_) synthesis (MAP3772c), and a ribosomal protein (rpmE2) coded in the other direction (24).

**TABLE 1:** Genes enriched in the presence of mycobactin J^*a*^

Gene name	Function	LSP^P^
*MAP0282c*	H.P.	
*MAP0900*	Membrane protein	
*MAP1155*	PPE family protein	
*MAP1420*	Peptide synthetase	
*MAP2525c*	Cytochrome P450	
*MAP3429*	Purine nucleoside phosphorylase	
*MAP3648c*	H.P.	
*MAP3740*	Nonribosomal peptide synthase	14
*MAP3742*	Thioester reductase	14
*MAP3764c*	Polyketide synthase Pks2	14
*MAP3770*	Cobalamin synthesis protein	
*MAP3775c*	ABC transporter, ATP-binding protein	15
*MAP3834*	Sugar transport protein	
*MAP3871*	Phosphoribosylglycinamide formyltransferase 2	
*MAP3883c*	Beta-lactamase	
*MAP3884*	F420-dependent glucose-6-phosphate dehydrogenase	
*MAP4076*	Glycosylhydrolase family protein	

a Genes identified to be enriched (5× median) in the presence of mycobactin J in two independent transposon screening experiments. Gene annotation was performed by Li et al. (19). H.P., hypothetical protein.

To tease out the functional significance of LSP^P^15, we then set out to isolate transposon mutants harboring a disruption(s) within this region. We screened 5,000 transposon mutants and isolated one clone that was PCR positive when the *MAP3775c* primer was used in combination with a transposon-specific primer, indicating a transposon mutant of *MAP3775c* or a nearby gene. Sequencing of the PCR product and the excised transposon-containing plasmid revealed that the transposon sequence was inserted at nucleotide position 4219504 in the genome, inside *MAP3776c* at nucleotide position 597 (see Fig. S2 in the supplemental material). This transposon mutant is denoted *MAP3776c*::Tn here. *MAP3776c* is predicted to encode the substrate-binding protein of an ABC transporter involved in the uptake of ferric siderophores and ions of metals such as iron, manganese, copper, and/or zinc (http://www.ncbi.nlm.nih.gov/gene/2718452).

### The *MAP3776c*::Tn mutant shows increased iron uptake.

**FIG 3:**
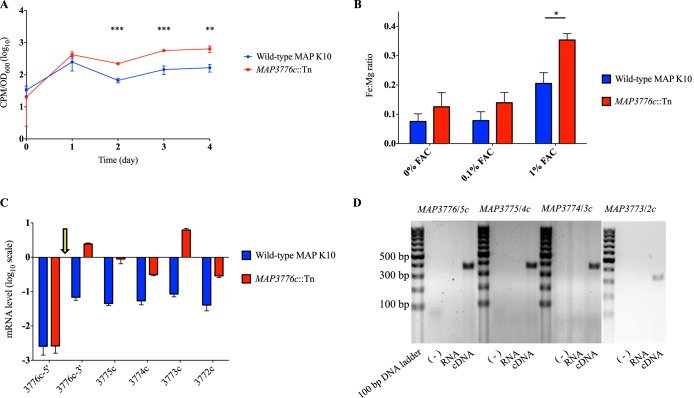
Characterization of LSP^P^15. (A) ^55^FeCl_3_ uptake assay in wild-type M. avium subsp. paratuberculosis and *MAP3776c*::Tn. **, *P* < 0.01; ***, *P* < 0.001. (B) ICP-MS quantification of intracellular metal contents (Fe:Mg) of strains grown in the presence of 0%, 0.01%, and 1.0% FAC for 48 h. Data are presented as means ± standard deviations of the results of two individual experiments, each performed in triplicate. *, *P* < 0.05 (compared with wild-type M. avium subsp. paratuberculosis intracellular Fe:Mg ratio). (C) Transcriptional analysis of LSP^P^15 genes in wild-type M. avium subsp. paratuberculosis and *MAP3776c*::Tn, quantified by qRT-PCR. Data shown are normalized to the expression of *sigA*, an endogenous housekeeping gene. The arrow indicates the transposon insertion. (D) Detection of cotranscription by amplifying the junction of each gene pair by PCR. Wild-type cDNA was used as a template; water and RNA served as negative controls. Lanes corresponding to reactions performed with each set of primers are separated by 100-bp DNA ladders (Thermo Scientific).

To test whether LSP^P^15 is involved in iron uptake, we compared ^55^FeCl_3_ uptake levels of wild-type M. avium subsp. paratuberculosis and the *MAP3776c*::Tn mutant. To our surprise, the transposon mutant exhibited a 3-fold increase in iron accumulation over the course of 4 days (Fig. 3A). To verify this finding, we exposed wild-type M. avium subsp. paratuberculosis and *MAP3776c*::Tn to 7H9 liquid broth supplemented with 0%, 0.1%, and 1.0% ferric ammonium citrate (FAC) and quantified their intracellular metal contents using inductively coupled plasma mass spectrometry (ICP-MS). As the bacterial magnesium transporters identified to date, such as those encoded by the *mgt* locus and *corA*, are not metal-binding ABC transporters like the one putatively encoded by *MAP3776-4c* (39–41), we reasoned that intracellular magnesium levels would remain stable regardless of our genetic manipulation in LSP^P^15 and could be used to normalize the iron contents (26). Analysis by ICP-MS revealed that *MAP3776c*::Tn showed a 1.7-fold-higher intracellular Fe:Mg ratio than wild-type M. avium subsp. paratuberculosis in 1.0% FAC (Fig. 3B). These observations were, however, contrary to our original intuition that disruption of LSP^P^15 would lead to a deficiency in iron uptake.

### LSP^P^15 genes are cotranscribed.

When we examined the transcriptional levels of the LSP^P^15 genes in the *MAP3776c*::Tn mutant, we found that genes *MAP3775c* through *MAP3772c* downstream of the transposon insertion were upregulated (Fig. 3C). The upregulation of these genes led us to test whether they are cotranscribed. Using wild-type M. avium subsp. paratuberculosis cDNA as the template, PCR with primers flanking the junction of neighboring genes produced amplicons for *MAP3776c* and *MAP3775c*, *MAP3775c* and *MAP3774c*, *MAP3774c* and *MAP3773c*, and *MAP3773c* and *MAP3772c*, consistent with a single transcript spanning these multiple genes (Fig. 3D). In addition, for wild-type M. avium subsp. paratuberculosis, we noticed low levels of mRNA corresponding to the predicted 5′ end of *MAP3776c*, in contrast to the levels measured for the 3′ end of the same gene. In the M. avium subsp. paratuberculosis K10 genome, *MAP3776c* is predicted to be 1,110 bp long, encoding a protein 369 amino acids in length. In MAP-sheep (S397 [38]), the MAP3776c protein is predicted to be only 263 amino acids in length and starts at position 107 relative to the K10 protein (see Fig. S3 in the supplemental material). To elucidate the correct start site, we used primers amplifying regions downstream of 4 putative start codons (GTG or ATG; nucleotide positions 1, 115, 319, and 654) and wild-type cDNA as the template, and PCR products were detected only with primer pairs that amplified regions downstream of the 3rd and 4th putative start codons, consistent with the S397 annotation (data not shown). To more precisely determine the transcription start site, we performed 5′ rapid amplification of cDNA ends (5′ RACE) and revealed that transcription starts 50 bp upstream of the 3rd predicted start codon (see Fig. S4 and S5).

### Upregulation of LSP^P^15 leads to increased iron uptake.

**FIG 4:**
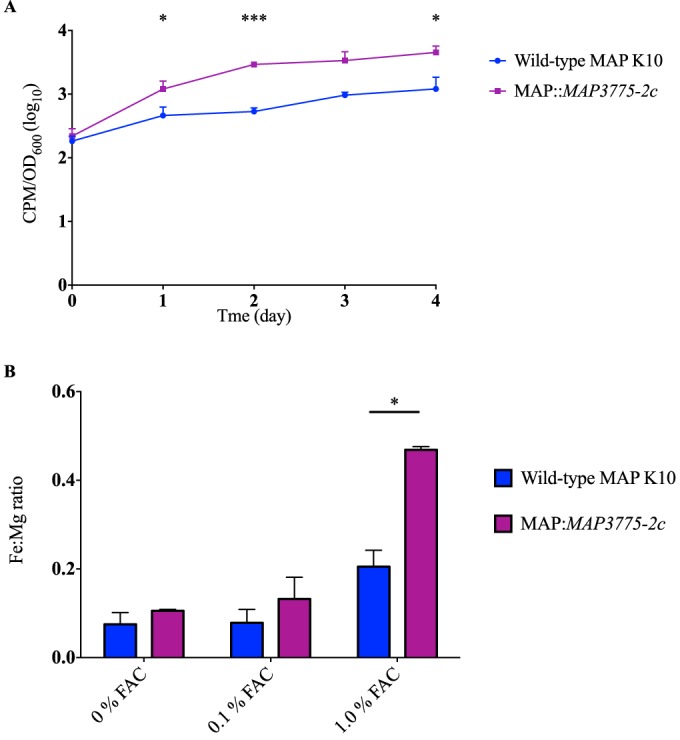
Functional consequences of LSP^P^15 overexpression. (A) ^55^FeCl_3_ uptake assay in wild-type M. avium subsp. paratuberculosis and MAP::*MAP3775-2c*. *, *P* < 0.05; ***, *P* < 0.001. (B) ICP-MS quantification of intracellular metal contents (Fe:Mg) of wild-type M. avium subsp. paratuberculosis and MAP::*MAP3775-2c* grown in 7H9 supplemented with 0%, 0.1%, or 1.0% FAC after 48 h. Data are presented as means ± standard deviations of the results of two individual experiments, each performed in triplicate. *, *P* < 0.05 (compared with wild-type M. avium subsp. paratuberculosis intracellular Fe:Mg ratio).

As the expression data indicated overexpression of other genes in the operon, we then hypothesized that *MAP3775c* and *MAP3774c*, predicted to encode the ATP-binding and transmembrane domains of an ABC transporter, respectively, could function without MAP3776c, the solute-binding protein, when free ferric ions were present in the environment. To test this possibility, we overexpressed *MAP3775-2c* in the wild-type strain. The resultant clones exhibited increased ^55^FeCl_3_ uptake as well as higher intracellular Fe:Mg ratios in 1.0% FAC, as analyzed by ICP-MS (Fig. 4). Together, these findings both explained why the Tn mutant manifests increased iron uptake and confirmed that proteins encoded by M. avium subsp. paratuberculosis-specific LSP^p^15 island contribute to iron acquisition by M. avium subsp. paratuberculosis.

## DISCUSSION

The truncation of *mbtA* has been proposed to be the underlying defect responsible for the mycobactin dependency observed in MAP-cattle in the laboratory (19). Here we showed that, although MAP-sheep does not share this *mbtA* truncation, neither sublineage synthesizes the same mycobactin species as M. intracellulare and other M. avium subspecies. Aside from the *mbt* genes, other nonribosomal peptide synthetases (NRPSs) may be encoded by the M. avium subsp. paratuberculosis genome, potentially accounting for the production of ^14^C salicylic acid-labeled derivatives as revealed by TLC. The capacity of these radiolabeled species to transport iron as indicated by iron uptake assays, however, appears to be negligible. Interestingly, *MAP3740* to -3746 within LSP^P^14 are predicted to be involved in siderophore biosynthesis and to encode proteins containing adenylation, condensation, and epimerization domains, typically seen in NRPSs (42, 43). Several mutants within this locus were found to be enriched in the presence of mycobactin J (Table 1).

A closer *in silico* examination of the *mbtE* gene revealed a common 31-bp deletion across MAP-cattle and -sheep strains as well as a second deletion unique to MAP-sheep. As deletion of *mbtE* in M. tuberculosis and M. smegmatis has been shown to abrogate mycobactin synthesis (14, 44), a deletion in *mbtE* would be likely to contribute to the inability of affected strains of M. avium subsp. paratuberculosis to produce mycobactin. Consistent with this, the genome of a mycobactin-independent strain of MAP-cattle revealed an intact *mbtE* gene, raising the possibility that some strains of M. avium subsp. paratuberculosis have not suffered this deletion (45). A more complete understanding of the potential role of *mbtE* in the loss of mycobactin synthesis will emerge when there are more genome sequences available for evaluation of the phylogenetic distribution of the *mbtE* deletion. Given that the *mbt* operon is regulated by the iron-dependent repressor protein IdeR in an iron-dependent and strain-specific manner (46, 47), defining the contribution of the individual *mbt* operon mutations to the loss of mycobactin synthesis will require defined deletion mutants and/or in *trans* complementation experiments. Although mycobactin production is crucial for iron acquisition in M. tuberculosis, some mycobacterial species, including M. haemophilum, M. leprae, and M. lepromatosis, have been found to harbor extensive deletions in the biosynthesis pathway, suggesting that alternative mechanisms for iron uptake may exist in these species (48–50). As the synthesis and utilization of mycobactins are complex processes in mycobacteria (9, 44), we speculate that the acquisition of an alternative iron uptake mechanism(s) may have occurred first, obviating the need to maintain the potentially more energetically expensive siderophore-based system. The fact that newly isolated M. avium subsp. paratuberculosis bacteria grow better *in vitro* in the presence of exogenous mycobactin (51) is congruent with our observation in this study and argues against the ablation of mycobactin biosynthesis as a consequence of a defective utilization pathway.

Alternative iron-scavenging strategies, such as that represented by the presence of an extracellular ferric reductase (21) and an inorganic metal transporter (*MAP3734-6c*) located in LSP^P^14 (52), have been proposed. Using an unbiased, transposon-mediated mutagenesis approach, we identified *MAP3775c* to be conditionally essential for growth of M. avium subsp. paratuberculosis in the absence of exogenous mycobactin. Genes in LSP^P^15 are differentially regulated in bovine tissues, and disruption in *MAP3776c* reduces bacterial fitness by 10-fold in the mouse, providing evidence for their physiologic relevance during infection (28, 53). We subsequently investigated the functional significance of LSP^P^15 in M. avium subsp. paratuberculosis iron metabolism and provided evidence that LSP^P^15 is involved in iron uptake.

Compared to LSP^P^14 (65.1 kb), the largest M. avium subsp. paratuberculosis-specific genomic island that potentially encodes multiple iron acquisition units (24), LSP^P^15 (5.4 kb) contains only 6 genes, putatively encoding an ABC transporter, a FurB/Zur-like transcription regulator, a cobalamin synthase, and a ribosomal protein (rpmE2). MAP3776c, the substrate-binding protein, has a TroA-like domain which is known to play a role in the binding of divalent metals such as iron, zinc, and manganese through direct interactions (54, 55). MAP3775c has a nucleotide-binding domain similar to that of ZnuC, the ATPase subunit of an ABC transporter, found in Escherichia coli (54, 56). *MAP3774c* encodes the transmembrane portion homologous to ZnuB (54). *MAP3776-4c* (as well as *MAP3773c*, a putative metal uptake regulator) has recently been found to be upregulated during zinc starvation (57). MAP3772c contains a cobalamin (vitamin B_12_) synthesis protein *cobW* C-terminal domain. *MAP3776c*, the first gene of this genomic island, appears to be misannotated, as its transcription start site (TSS) was shown to be 268 bp downstream of the predicted start site. The 50-bp sequence upstream of our identified TSS likely represents the 5′ untranslated region, which is not uncommon and can range from 0 to 500 bp in length in mycobacteria (58, 59).

Using transposon mutagenesis, we were able to disrupt the first gene in the LSP^P^15 region and showed that this 5-gene locus is cotranscribed as an operon, commonly seen in horizontally transferred genomic islands (60–62). Because of this cotranscription, the transposon insertion in *MAP3776c* resulted in the overexpression of *MAP3775-2c* due to the presence of a strong promoter driving the expression of antibiotic resistance within the transposon sequence (63, 64). Consequently, by both ^55^FeCl_3_ uptake assay and ICP-MS, the transposon mutant exhibited higher intracellular iron content, a phenotype also seen by overexpressing *MAP3775-2c* under the control of a constitutive promoter in the wild-type strain. Together, these findings indicate that the genes *MAP3775-2c* contribute to iron acquisition by M. avium subsp. paratuberculosis.

The regulation of LSP^P^15 is not well understood. Neither IdeR nor the ferric uptake regulator (FurA), both important in regulating iron metabolism in M. tuberculosis (65, 66), appears to regulate LSP^P^15 genes in M. avium subsp. paratuberculosis (47, 67). The FurB transcription regulator, predicted to be encoded by *MAP3773c*, has been implicated in zinc homeostasis in M. tuberculosis and M. avium subsp. paratuberculosis (57, 68). Although its expression was not altered in wild-type M. avium subsp. paratuberculosis grown in different concentrations of iron (data not shown), its transcript was present at higher levels in the *MAP3776c*::Tn mutant grown in regular 7H9 compared to wild-type M. avium subsp. paratuberculosis. Whether the overexpression of *MAP3773c* has any impact on LSP^P^15 or the core genome is under investigation.

Previous studies have suggested that M. avium subsp. paratuberculosis-specific genomic islands are important for bacterial survival inside mammalian hosts (28, 69). Through bioinformatic prediction and experimentation, the current study demonstrated that LSP^P^15 may provide an alternative iron uptake system. From an evolutionary standpoint, it is of immense interest to observe the plasticity of the M. avium subsp. paratuberculosis genome and to understand the interplay between the ancestral core genome and LSP^P^15, and other horizontally transferred genomic islands, in this host-adapted pathogenic clone of M. avium.

## Supplementary Material

Supplemental material
