# Supplementary material for: Iron Acquisition in Mycobacterium avium subsp. paratuberculosis
Source: J Bacteriol. 2016 Feb 12;198(5):857–66. doi: 10.1128/JB.00922-15 (PMC4810606; doi:10.1128/JB.00922-15)
Supplement: Supplemental material [file supp_198_5_857__index.html]

Supplemental material 

# Iron Acquisition in Mycobacterium avium subsp. paratuberculosis

## Supplemental material

- Supplemental file 1 -

  Fig. S1, multiple alignment of *mbtE* sequences

  Fig. S2, sequence of *MAP3776c* disrupted by a transposon

  Fig. S3, comparison of MAP3776c protein with S397

  Fig. S4, transcription start site of *MAP3776c*

  Fig. S5, partial sequence chromatogram showing fusion of RNA oligonucleotide and *MAP3776c*

  PDF, 717K
- Supplemental file 2 -

  Table S1, primers

  Table S2, ICP-MS raw data

  Table S3, libraries of enriched genes

  XLSX, 51K
