# Supplementary material for: Iron Acquisition in Mycobacterium avium subsp. paratuberculosis
Source: J Bacteriol. 2016 Feb 12;198(5):857–66. doi: 10.1128/JB.00922-15 (PMC4810606; doi:10.1128/JB.00922-15)
Supplement: Supplemental material [file JB.00922-15_zjb999093954so1.pdf]

Fig. S1

A.

```
MAH_mbtE        gcgctggtggaccgcgacgacgtggacccgcgggcgggccgactgcgaccgccccacccc    5100
MAPc_mbtE       GCGCTGGTGGACC-------------------------------GCGACCGCCCCACCCC    5069
MAPs_mbtE       gcgctggtggacc-------------------------------gcgaccgccccacccc    4917
                *************                               ****************
```

B.

```
MAH_mbtE        cgcgtgcagtacgccgactacgcggcctggcagcgcacgtttctcggtgacgctggcggc    3780
MAPc_mbtE       CGCGTGCAGTACGCCGACTACGCGGCCTGGCAGCGCACGTTTCTCGGTGACGCCGGCGGC    3780
MAPs_mbtE       cgcgtgcagtacgccgactacgcggcctggcagcgcacgtttctcggtgacgccggcg--    3778
                ***************************************************** ****

MAH_mbtE        caggagtccgcggttgccggtgcgcagcgcgagtactggacccggcagctggccgggctg    3840
MAPc_mbtE       CAGGAGTCCGCGGTTGCCGGTGCGCAGCGCGAGTACTGGACCCGGCAGCTGGCCGGGTTG    3840
MAPs_mbtE       ------------------------------------------------------------    3778

MAH_mbtE        ccggaggacaccgggctgcgcccggacttcccgcgccagccggtgcccagcggggacggc    3900
MAPc_mbtE       CCGGAGGACACCGGACTGCGTCCGGACTTCCCGCGCCAGCCGGTGCCCAGCGGGGACGGC    3900
MAPs_mbtE       ------------------------------------------------------------    3778

MAH_mbtE        gaatccgtcgacttccacatcgacgcggccaccagggccaagctcgccgacgtgtgccgc    3960
MAPc_mbtE       GAATCCGTCGACTTCCACATCGACGCGGCCACCAGGGTCAAGCTCGCCGAGGTGTGCCGC    3960
MAPs_mbtE       ------------------------------gccagggtcaagctcgccgaggtgtgccgc    3808
                               ****** ************ *********
```

Multiple alignment of *mbtE* nucleotide sequences. Alignment was generated using Clustal Omega using default settings on Oct 31, 2015.

A) Nucleotide sequence showing common deletion event at 5,053 in MAP-cattle and -sheep genomes, relative to the MAH genome

B) Nucleotide sequence showing deletion event at 3,778 specific to the MAP-sheep genome, relative to the MAH and MAP-cattle genomes.

Fig. S2

START →
4220100
atggcgacggtgggcgcatgttgccgctcgggcgggccagccggggtcccgcggaaccgggccggcgcgcaccgcgacgcggaccgcgtttccggaa
agtctcgcgcattagatatgaaaacggttatcgtttgcggtgtgggtgggttgctctcgcggtggcacggcgcccctatcgctactgtcctggtgctgacagtcgt
aacgagttgctcgtcaagcccgacccaaacagccgaggggtcccgtaatgccgcctcgccatcagccattggcagaacggccgcgccgccgtgtcccac
cgcccccactcgctgtcgtggtcagcgtggatcagtggggcgacattgtctccgagctgggcggcgcctgcgcgaacgtcaagaccgtgctcgccagctcgtc
ggtcgacccgcacgattacgagccgtcaccggccgacgccgctgactttatgaacgcgaaacttatcgtggtcaacggtgcagggtacgactcgtgggcat
ccaagctggccggcagctccgcttccggtgccccccttggtgagcgcccgcggcagtcacgacaacaccggatggagccaaccctcacctgtgg
4219501
ACAGGTTGGCTGATAAGTCCCCGGTC*TCTAGACCCCTATAGTGAGTCGT*ATTACAAACTGGAACAACACTC
AACCCTATCTCGGTCTATTCTTTTGATTTATAAGGGATTTTGCCGATTTCGGCCTATTGGTTAAAAAATGAGC
TGATTTAACAAAAATTTAACGCGAATTTTAACAAAATATTAACGCTTACAATTTAGGTGGCACTTTTCGGGGA
AATGTGCGCGGAACCAGATCCCCACGATGCGTCCGGCGTAGAGGATCTGAAGATCAGCAGTTCAACCTGT
TGATAGTACGTACTAAGCTCTCATGTTTCACGTACTAAGCTCTCATGTTTAACGTACTAAGCTCTCATGTTTA
ACGAACTAAACCCTCATGGCTAACGTACTAAGCTCTCATGGCTAACGTACTAAGCTCTCATGTTTCACGTAC
TAAGCTCTCATGTTTGAACAATAAAATTAATATAAATCAGCAACTTAAATAGCCTCTAAGGTTTTAAGTTTTAT
AAGAAAAAAAAGAATATATAAGGCTTTTAAAGCTTTTAAGGTTTAACGGTTGTGGACAACAAGCCAGGGATG
TAACGCACTGAGAAGCCCTTAGAGCCTCTCAAAGCAATTTTCAGTGACACAGGAACACTTAACGGCTGACA
GAATTCTTGAAGACGAAAGGGCCTCGTGATACGCCTATTTTTATAGGTTAATGTCATGATAATAATGGTTTC
TTAGACGTCAGGTGGCACTTTTCGGGGTTATAAGGGCTGCAGGAATTCGATATCAAGCTTATCGATACCGT
CGACCTCGAGGGGGGGCCCGGTACCGAGGACGCGTCGAATTAATTCCGCTAGCTTCACGCTGCCGCAAG
CACTCAGGGCGCAAGGGCTGCTAAAGGAAGCGGAACACGTAGAAAGCCAGTCCGCAGAAACGGTGCTGA
CCCCGGATGAATGTCAGCTACTGGGCTATCTGGACAAGGGAAAACGCAAGCGCAAAGAGAAAGCAGGTAG
CTTGCAGTGGGCTTACATGGCGATAGCTAGACTGGGCGGTTTTATGGACAGCAAGCGAACCGGAATTGCC
AGCTGGGGCGCCCTCTGGTAAGGTTGGGAAGCCCTGCAAAGTAAACTGGATGGCTTTCTTGCCGCCAAGG
ATCTGATGGCGCAGGGGATCAAGATCTGATCAAGAGACAGGATGAGGATCGTTTCGCATGATTGAACAAG
ATGGATTGCACGCAGGTTCTCCGGCCGCTTGGGTGGAGAGGCTATTCGGCTATGACTGGGCACAACAGAC
AATCGGCTGCTCTGATGCCGCCGTGTTCCGGCTGTCAGCGCAGGGGCGCCCGGTTCTTTTTGTCAAGACC
GACCTGTCCGGTGCCCTGAATGAACTCCAAGACGAGGCAGCGCGGCTATCGTGGCTGGCCACGACGGGC
GTTCCTTGCGCAGCTGTGCTCGACGTTGTCACTGAAGCGGGAAGGGACTGGCTGCTATTGGGCGAAGTGC
CGGGGCAGGATCTCCTGTCATCTCACCTTGCTCCTGCCGAGAAAGTATCCATCATGGCTGATGCAATGCG
GCGGCTGCATACGCTTGATCCGGCTACCTGCCCATTCGACCACCAAGCGAAACATCGCATCGAGCGAGCA
CGTACTCGGATGGAAGCCGGTCTTGTCGATCAGGATGATCTGGACGAAGAGCATCAGGGGCTCGCGCCA
GCCGAACTGTTCGCCAGGCTCAAGGCGCGGATGCCCGACGGCGAGGATCTCGTCGTGACCCATGGCGAT
GCCTGCTTGCCGAATATCATGGTGGAAAATGGCCGCTTTTCTGGATTCATCGACTGTGGCCGGCTGGGTG
TGGCGGACCGCTATCAGGACATAGCGTTGGCTACCCGTGATATTGCTGAAGAGCTTGGCGGCGAATGGG
CTGACCGCTTCCTCGTGCTTTACGGTATCGCCGCTCCCGATTCGCAGCGCATCGCCTTCTATCGCCTTCTT
GACGAGTTCTTCTGAGCGGGACTCTGGGGTACGCG*TAATACGACTCACTATAGGG*TCTAGAGACCGGGGA
CTTATCAGCCAACCTGT
4219502
tacctgcccctccgcggtgaccgccgttgctgatgcggtaacgcaagaattgagcagaatggaaccgcctgccgccggctacttcagccagcggagagcgc
aattcaccagtgcgacaaggctatacgtgaacctgatcgccaagatcaaggccgaggcggcgggtaaatcctatggggccacagagacggtctttgacta
ccaggcgcaagcggcgggcctggtcaacaagacccctgccgggtaccggcgggcatcggccaacgaatcggaaccctcacccggagacgttgacgcg
ttcctcaccgccccttgccggccggcatatcgacctcttgatctacaacacgcagaccgagggctcaatccccgaggaaatccgatcagccgcggagcagtc
aagcgtgcccgtcgtcaagataaccgagaccgtaccgcccggggagacgtcgtttgaagactggcagtacgggcagctcgtccaactggccaaggcgct
ccatgtcgctgtctga
4218991
END

The sequence of *MAP3776c* disrupted by the ΦMycoMarT7 transposon.
The entirety of the *MAP3776c* gene containing the transposon is shown. Lower-case letters indicate nucleotides from *MAP3776c*, while the upper-case letters indicate nucleotides from the transposon. Numbers indicate the standard nucleotide number in MAP. Highlighting shows the features of the transposon: blue = inverted Himar repeats; gray = ori6K, yellow = neomycin phosphotransferase. Red, italicized letters show outward-facing T7 promoter sites. The Genbank accession numbers for *MAP3776c* and the transposon are AE016958.1 and AF411123, respectively (adapted from (61)).

Fig. S3

ABC-type metal ion transport system, periplasmic component/surface adhesin [Mycobacterium avium subsp. paratuberculosis S397]
Sequence ID: gb|EGO39095.1| Length: 263 Number of Matches: 1

Range 1: 1 to 263 GenPept Graphics ▼ Next Match ▲ Previous Match

Score	Expect	Method	Identities	Positives	Gaps
530 bits(1364)	0.0	Compositional matrix adjust.	261/263(99%)	262/263(99%)	0/263(0%)

```
Query  107  VVSVDQWGDIVSELGGACANVKTVLASSSVDPHDYEPSPADAADFMNAKLIVVNGAGYDS  166
            +VSVDQWGDIVSELGGACANVKTVLASSSVDPHDYEPSPADAADFMNAKLIVVNGAGYDS
Sbjct  1    MVSVDQWGDIVSELGGACANVKTVLASSSVDPHDYEPSPADAADFMNAKLIVVNGAGYDS  60

Query  167  WASKLAGSSASGAPLVSAAAVTTTPDGANPHLWYLPSAVTAVADAVTQELSRMEPPAAGY  226
            WASKLAGSSASGAPLVSAAAVTTTPDGANPHLWYLPSAVTAVADAVTQELSRMEPPAAGY
Sbjct  61   WASKLAGSSASGAPLVSAAAVTTTPDGANPHLWYLPSAVTAVADAVTQELSRMEPPAAGY  120

Query  227  FSQRRAQFTSATRLYVNLIAKIKAEAAGKSYGATETVFDYQAQAAGLVNKTPAGYRRASA  286
            FSQRRAQFTSATRLYVNLIAKIKAEAAGKSYGATETVFDYQAQAAGLVNKTPAGYRRASA
Sbjct  121  FSQRRAQFTSATRLYVNLIAKIKAEAAGKSYGATETVFDYQAQAAGLVNKTPAGYRRASA  180

Query  287  NESEPSPGDVDAFLTALAGRHIDLLIYNTQTEGSIPEEIRSAAEQSSVPVVKITETVPPG  346
            NESEPSP DVDAFLTALAGRHIDLLIYNTQTEGSIPEEIRSAAEQSSVPVVKITETVPPG
Sbjct  181  NESEPSPRDVDAFLTALAGRHIDLLIYNTQTEGSIPEEIRSAAEQSSVPVVKITETVPPG  240

Query  347  ETSFEDWQYGQLVQLAKALHVAV  369
            ETSFEDWQYGQLVQLAKALHVAV
Sbjct  241  ETSFEDWQYGQLVQLAKALHVAV  263
```

Comparison of the *M. avium* subsp. *paratuberculosis* K-10 (Query) MAP3776c protein with that of S397 (Sbjct). Alignment was generated by Protein BLAST. Last accessed July 5, 2015

Fig. S4

Predicted transcript (KEGG) – black

Proposed transcript (our work/MAP-sheep)– red

Blue – transcript start site identified in this RACE experiment

atggcgacggtgggcgcatgttgccgctcgggcgggccagccggggtcccgcggaaccgg
gccggcgcgcaccgcgacgcggaccgcgtttccggaaagtctcgcgcattagatatgaaa
acggttatcgtttgcggtgtgggtgggttgctctcgcggtggcacggcgcccctatcgct
actgtcctggtgctgacagtcgtaacgagttgctcgtcaagcccgacccaaacagccgag
gggtcccgtaatgccgcctcgccatcagccattggcagaacggccgcgccgccgtgtccc
accgccccactcgctgtcgtggtcagcgtggatcagtggggcgacattgtctccgagctg
ggcggcgcctgcgcgaacgtcaagaccgtgctcgccagctcgtcggtcgacccgcacgat
tacgagccgtcaccggccgacgccgctgactttatgaacgcgaaacttatcgtggtcaac
ggtgcagggtacgactcgtgggcatccaagctggccggcagctccgcttccggtgccccc
ttggtgagcgccgcggcagtcacgacaacaccggatggagccaaccctcacctgtggtac
ctgccctccgcggtgaccgccgttgctgatgcggtaacgcaagaattgagcagaatggaa
ccgcctgccgccggctacttcagccagcggagagcgcaattcaccagtgcgacaaggcta
tacgtgaacctgatcgccaagatcaaggccgaggcggcgggtaaatcctatggggccaca
gagacggtctttgactaccaggcgcaagcggcgggcctggtcaacaagacccctgccggg
taccggcgggcatcggccaacgaatcggaaccctcacccggagacgttgacgcgttcctc
accgcccttgccggccggcatatcgacctcttgatctacaacacgcagaccgagggctca
atccccgaggaaatccgatcagccgcggagcagtcaagcgtgcccgtcgtcaagataacc
gagaccgtaccgcccggggagacgtcgtttgaagactggcagtacgggcagctcgtccaa
ctggccaaggcgctccatgtcgctgtctga

Transcription start site of *MAP3776c* identified by 5' RACE.

Fig. S5

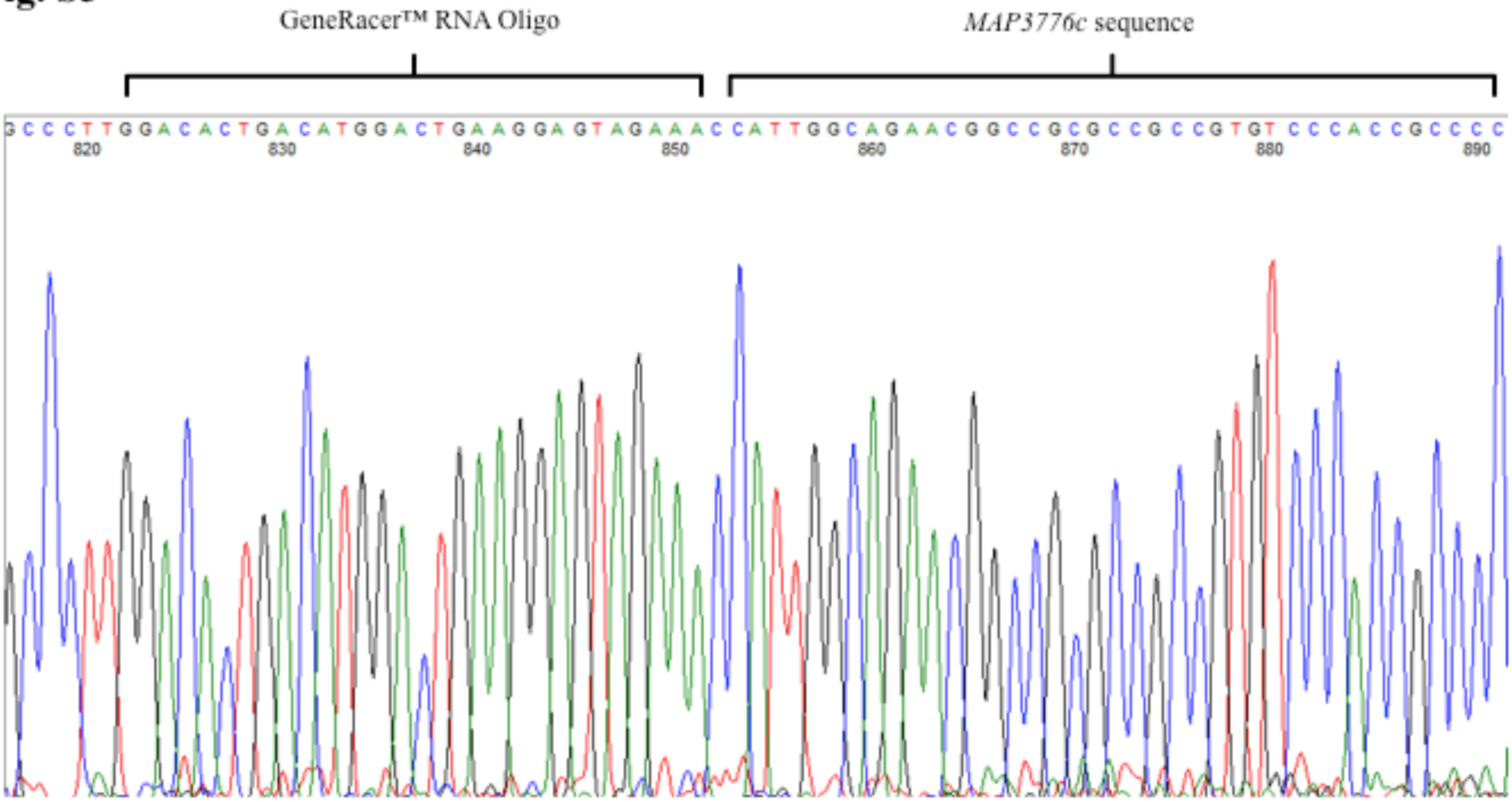

Partial sequence chromatogram of 5'-RACE showing the fusion of RNA oligo and *MAP3776c*. Visualized with FinchTV (http://www.geospiza.com/Products/finchtv.shtml).
